# A retrospective diagnostic study of prevalence of orofacial calcifications using panoramic radiograph: To insinuate the unseen

**DOI:** 10.4317/jced.60224

**Published:** 2023-04-01

**Authors:** Annapoorani S., Jones-Rajadeva Thambi, Winnifred Christy

**Affiliations:** 1 Intern,CSI College of Dental Sciences and Research, Madurai, India; 2 Reader, Department of Oral Medicine and Radiology, CSI College of Dental Sciences and Research, Madurai, India; 3 Professor and Head, Department of Oral Medicine and Radiology, CSI College of Dental Sciences and Research, Madurai, India

## Abstract

**Background:**

The prevalence of calcifications in the head and neck region has long been observed and has a strong value of presaging systemic illness. The observations of such calcifications in routine panoramic radiographs (PR) demands keen follow-up and health check-up of patients. In developing countries, the use of routine panoramic radiographs is a common one owing to its cost effectiveness and feasibility. Thus, knowledge of prevalent calcifications and the ability to diagnose it while correlating with possible systemic condition is mandatory. This article is primarily about the prevalence of soft tissue calcifications in head and neck region while emphasising the clinical importance.

**Material and Methods:**

A total of 22,000 panoramic radiographs were included after adapting the inclusion and exclusion criteria. All the included radiographs were examined using Dentsply Sirona Sidexis 4 Dental imaging software in full screens.

**Results:**

A total of 22,000 PRs were analyzed, of which 7,832 were male and 14,168 were female. The age range of patients included were from 6 to 88 years with a mean of 41 years ± 11.4 years standard deviation. Of the analyzed PR’s, a total of 1228 calcifications were found in 1041 (4.731%) patients which comprised of 497(6.34%%) calcifications in male and 731 calcifications in female (5.159%). From which, 16 different soft tissue calcifications were reported while stylomandibular ligament calcification being the most reported one.

**Conclusions:**

Panoramic radiographs is yet an essential diagnostic tool, as a dentist our role in diagnosing systemic conditions is inevitable. A high prevalence of calcifications demands thorough examination of radiographs on routine. Early detection od calcifications ensures prevention of further progression of disease.

** Key words:**Orofacial calcification, panoramic radiography, atherosclerosis, dystrophic calcification.

## Introduction

The deposition of calcium salts in tissues such as calcium phosphate, in sites other than osteoid/bone or hard tissues of teeth structure such as soft tissues in an unordered fashion is called Heterotopic or Pathologic calcification (1,2). Such Soft Tissue Calcifications (STC) are usually undiagnosed unless incidentally found during routine radiologic examination. There are three major types of STC, namely dystrophic, metastatic and idiopathic. They are classified mainly based on the serum calcium levels and the site where they get deposited. Dystrophic calcification occurs when the serum calcium levels are normal and seen mostly in dead and degenerated tissues. Metastatic calcification occurs when serum calcium and phosphate levels are elevated i.e.) deranged metabolism and hypercalcemia (1-4). Finally, idiopathic calcification also referred as calcinosis occurs in normal tissues in presence of normal calcium levels. STC in the head and neck region are uncommon and are mostly asymptomatic. However, the presence of some calcification indicates presence of serious underlying systemic disease which warrants definitive treatment. They are identified mostly based on their anatomic location, number, distribution, calcification pattern, size and shape (5).

The listing and classification for STC was drafted by White and Pharoah initially. It ranges from the most common stylohyoid ligament calcification, sialolith, lymph node calcification, myositis ossificans, Carotid artery calcification, antrolith and phlebolith. Epidemiologically, people above 40 years of age have high chances of developing pathologic calcifications and also there is a reported female predilection. Panoramic radiographs (PR) play a vital role in dental diagnostic procedures. They are 2-Dimensional radiograph which provides detailed visualisation of oral and para oral structures. During routine radiographic examination, STC may be detected as an incidental finding. Detection of such STC in PR is of greater significance and importance as they are early indicators of undiagnosed underlying systemic disease or an impending risk.

Thus, this study is mainly aimed to detect the STC during routine clinical examination by analysing Panoramic Radiographs (PR). Furthermore, this is the first kind of study to be done in South Indian population to include 16 different types of head and neck calcifications with added advantage of using a large sample size.

## Material and Methods

-Patient Assortment: This population based retrospective descriptive study was designed to evaluate the incidental findings of STC in PR of patients attending Oral Medicine and Radiology department between March 2017 to March 2022. A total of 22,000 PRs were assessed and evaluated. PR of all patients irrespective of age and gender were included in this study.

-Exclusion criteria.

• Faulty radiographs which include processing errors, imaging errors and patient positioning errors were not included.

• Images which didn’t extend up to styloid process above and hyoid bone below were excluded.

-Image acqusition: The PRs were taken using orthopantomographic device. The images were taken by specially trained radiograph technicians following proper guidelines and under ALARA (As Low As Reasonably Achievable) principle. Standard principles such as 66 kV, 5-10 mA for an exposure time of 17.6 seconds were followed while recording the radiographs.

**Figure 1:**
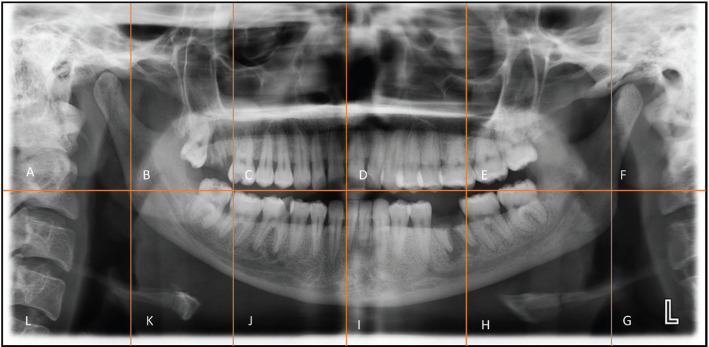
OPG segmenting.

-Image analysis: The acquired PR were assessed on full screen in a Dentsply Sirona Sidexis 4 Dental imaging software in full screens, the exposure of image was adjusted as needed to get a clear observation over the necessary areas. The radiopacities found in radiographs were assessed further for its size, shape, borders, number and location, either unilateral or bilateral. This be used further for risk assessment and to determine the prognosis of patients. Then PR divided into 12 sections by using one horizontal line across occlusal plane and 5 vertical lines along angle of mandible on both sides, through distal aspect of first molars on both sides and through midline as shown below were used as a guide for locating the position and for better recording (Fig. 1).

-Objectives:

• Prevalence of soft tissue calcification seen in PR.

• Estimate and compare the prevalence of soft tissue calcifications seen in male and females

• Estimate the most common age group affected

• Evaluate and compare the incidence of each calcification included

• Evaluation of most common location of occurrence of each calcification.

Find the relation between various factors associated with soft tissue calcification.

-Statistical analysis: IBM SPSS (Statistical Package for Social Sciences) was used. Spearman’s rank correlation was used to find the association between STC and various associated factors. Multinomial analysis is used to Figure out the most commonly affected age group in each type of STC.

-Soft tissue calcification assessed.

**Figure 2:**
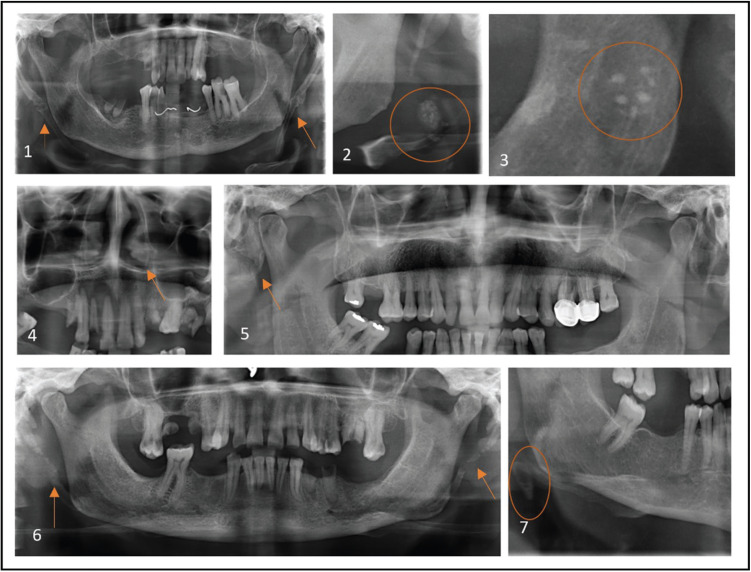
Various STC Observed. 1-Bilateral stylohyoid ligament calcification, 2- Carotid atherosclerotic plaque, 3- Multiple tonsiloliths at the level of mandibular ramus, 4- Bilateral antrolith, 5- Petrified ear adjacent to temporomandibular joint, 6- Bilateral, irregular stylomandibular ligament calcification, 7- Triticeous cartilage calcification.

**Figure 3:**
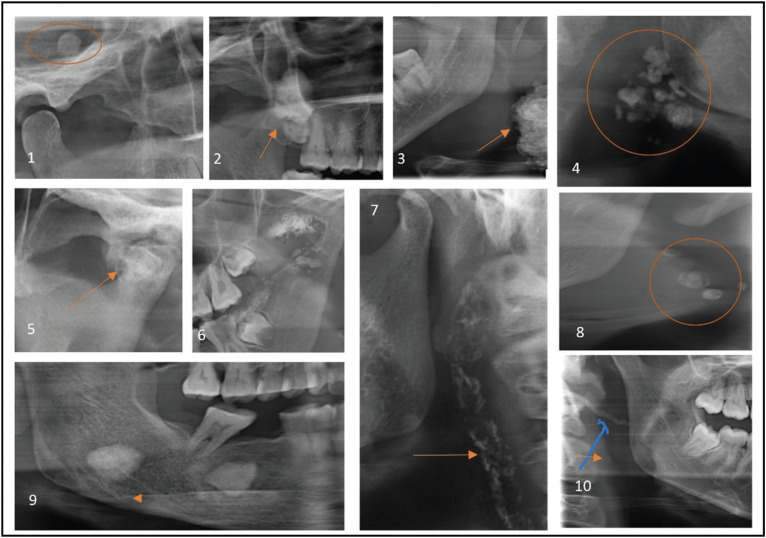
Soft tissue calcifications observed. 1-Superficial temporal artery calcification, 2- Antrolith, 3- Cervical lymph node calcification, 4- Multiple, irregular sum-mandibular lymph node calcification, 5- Temporo mandibular joint calcificatoin, 6- Myositis ossificas, 7- Monckeberg’s atherosclerosis of carotid artery, 8- Multiple phlebolith, 9- Submandibular sialolith, 10- Oro-pharyngeal calcification.

The STC included in this study is listed in Table 1,  Table 1 cont., Figs. 2,3.

**Table 1:**
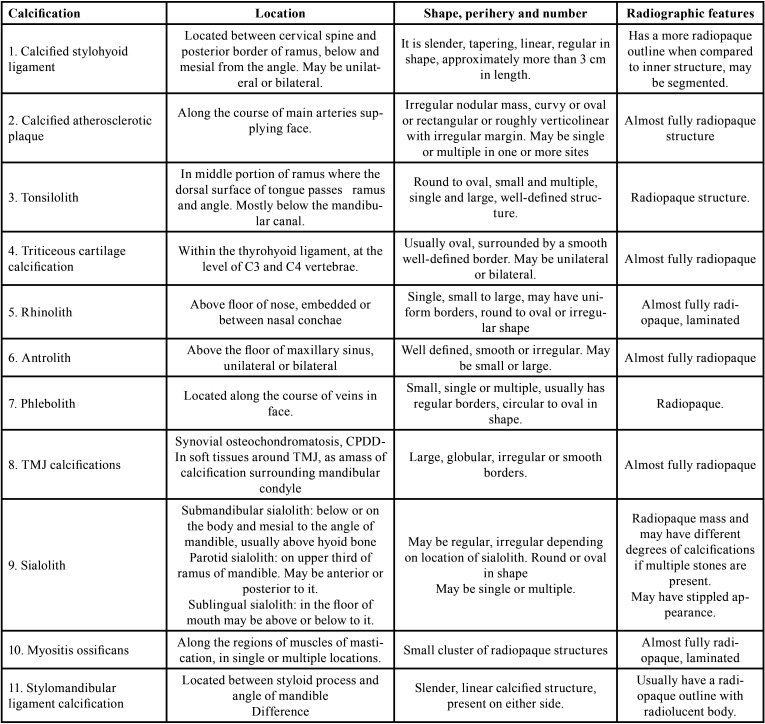
Soft Tissue Calcifications included.

**Table 1 cont.**
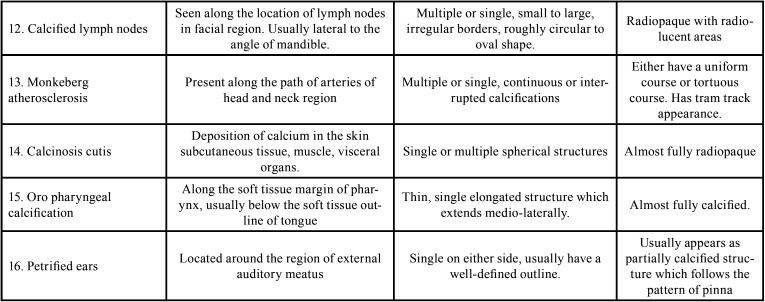
Soft Tissue Calcifications included.

## Results

A total of 22,000 PRs were analyzed, of which 7,832 were male and 14,168 were female. The age range of patients included were from 6 to 88 years with a mean of 41 years ± 11.4 years standard deviation. Of the analyzed PR’s, a total of 1228 calcifications were found in 1041 (4.731%) patients which comprised of 497(6.34%%) calcifications in male and 731 calcifications in female (5.159%). Apparently, more calcifications were found in male. The number of STC in each group is described in detail in Table below, categorized as male and female and further into the calcification level, appearance in unilateral or bilateral side (Table 2).

**Table 2:**
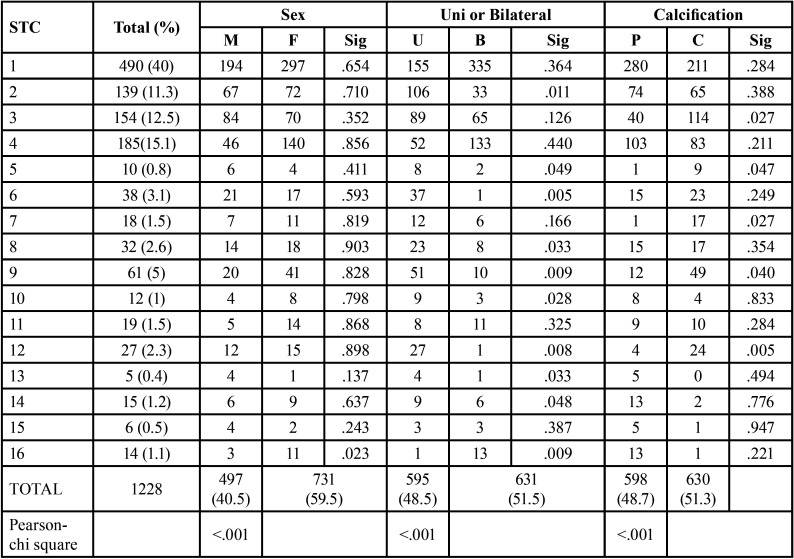
Pearson’s chi square test.

The most common STC identified was stylohyoid ligament calcification (490, 40%), followed by Triticeous cartilage calcification (185, 15.1%), tonsilolith (154, 12.5%), calcified atherosclerotic plaque (139, 11.3%). From independent t test, it resulted that calcified atherosclerotic plaque showed significantly unliteral than bilateral occurrence (*p*=.011), similarly rhinolith, antrolith, sialolith, calcified lymph nodes, Monkeberg atherosclerosis and calcinosis cutis occurs significantly unilaterally. Petrified ear showed a significant female predilection (p-.023). When considering the level of calcification, tonsilolith, rhinolith, sialolith, calcified lymph nodes show a statistically significant completely calcified structure.

Almost 90% of the incident sialolith were found in submandibular region, followed by sublingual and parotid region. Most of the encountered lymph node calcifications were of submandibular lymph nodes, only one case showed cervical node calcification. When looking into calcified atherosclerotic plaque, maximum was of carotid artery calcifications, few were superficial temporal artery calcification.

The most common region of occurrence of STC was L (164), followed by G (119) in cases when STC occurs unilaterally. When STC occurs bilaterally, the most common region of occurrence was L, G combined (326) and A, F combined (203) (Table 3).

**Table 3:**
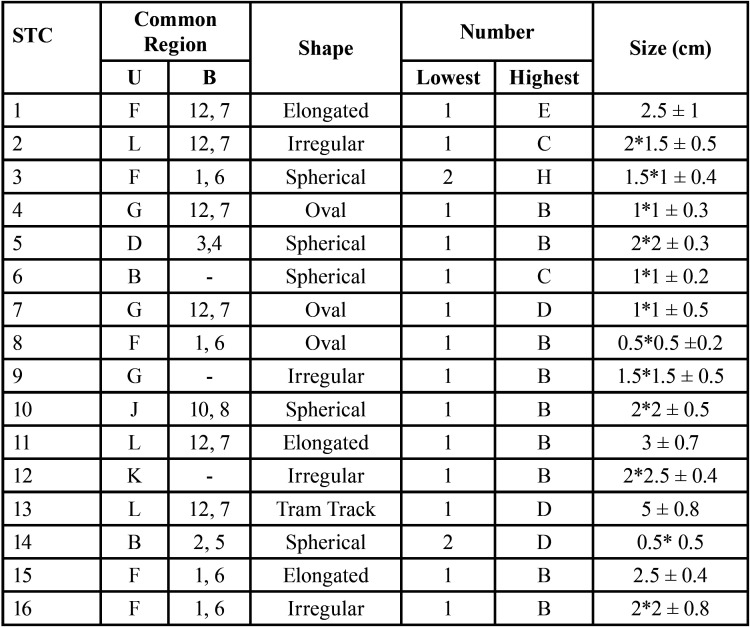
Characteristics of STC included.

A spearman’s rank correlation test was carried out to find the correlation of STC and its associated factors such as sex, age, level of correlation. This resulted in a significant correlation between age, calcification level and the side of occurrence of calcification (Table 4).

**Table 4:**
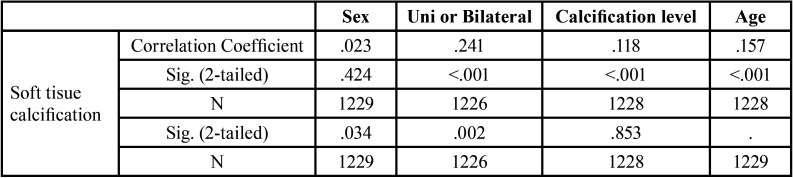
Spearman’s rank correlation for STC.

Here, no further assessment was made to correlate the systemic conditions of included patients with STC, rather this correlation was justified using literature evidences while highlighting the insinuation of STC in patients health.

## Discussion

Panoramic radiography is one of the common extra-oral diagnostic tools in dentistry, advised when there is a need to examine the oral cavity with extraoral structures. This study is mainly pertained to the evaluation and analysis of the STC encountered accidentally during routine radiographic examination. The sample size of 22,000 PR makes this study to more valuable and serves as a chance to gain additional information which might help to orient imminent investigations in this field.

The prevalence of soft tissue calcification of this study is 4.731%, which is in accordance with previous study reported by Mansour *et al*. (4%) (6). This level of incidence of STC is comparatively higher when compared to the studies conducted by Ivonne Garay (2.16%) (5). The mean age of patients with STC in this study was found to be 41 years ± 11.4 years. When looking into the most common region involved, region L, G (609, 49.5%) was commonly involved as most of the STC were localized in this region when they occur either unilaterally or bilaterally. In this study, a strong male predilection was noted. A total of 16 soft tissue calcifications were noted in this retrospective study which makes this to stand apart from other studies. Few of the rare STC of the oral and maxilla-facial regions such as petrified ear, oro-pharyngeal calcification, superficial temporal artery calcification and monckeberg’s atherosclerosis were reported in this study. It is a known fact that most of the reported STC have a less clinical significance but they are of no exception for severe outcomes, while few are indicators of severe underlying systemic disease. This warrants proper care at correct time to prevent any further complications. The highly prevalent STC encountered in present study is stylohyoid ligament calcification (490, 40%), followed by Triticeous cartilage calcification (185, 15.1%), tonsilolith (154, 12.5%), calcified atherosclerotic plaque (139, 11.3%).

Maximum of the calcified atherosclerotic plaques found were of carotid artery involvement, of which most were found in the region of carotid artery bifurcation (78.22%), few of them were found in the region of superficial temporal artery. Atherosclerotic plaques were irregular, partially calcified structures of size 1.5*1.5 CM. 58% of cases showed presence of single calcification while remaining cases showed presence of multiple calcifications, to the highest of 4 calcifications. PR remains to be a cost-effective way of finding the presence of carotid artery calcifications (7). The presence of calcifications in artery implies impending risk of developing complications. Patients with carotid artery calcifications are more prone to develop ischemic heart disease which is also linked to high risk or morbidity and mortality (8). When calcifications are in higher region as in superficial temporal artery, they are prone to develop intracranial ischemic disease (9) and more related to patients with chronic kidney disease. Additionally, it has been reported that patients with carotid plaque are in increased risk of developing peripheral vestibular disorder (PVD) (10). Masaoki Wada *et al*. in their study concluded that the hazard ratio of developing PVD is about 3.25 to 4.41.

Monckeberg arteriosclerosis is a disease characterized by dystrophic calcification of tunica media and internal elastic lamina ultimately leading to reduced arterial compliance. It has been associated with increased mortality and morbidity (11). This disease is usually of unknown etiology, sometimes maybe associated with hyperparathyroidism, coronary artery disease. In our study, a total of 5 cases were identified with a mean age of 45 years, male predilection was noted, this finding is in accordance with that of Kroger *et al*. findings. Middle aged male patients were most commonly affected. Radiographically, multiple tortuous vascular calcifications in the region adjacent to posterior border of mandibular ramus were noted. Few cases showed presence of a dense tram track appearance mostly of the maxillary and facial artery. A single case showed presence of carotid atherosclerotic plaque.

14 cases of petrified ears were also noted in this study. The mean age of reported patients were 55.5 years with a female predilection. Middle aged women were mostly affected. There was a significant bilateral involvement and most of the reported cases were partially calcified. Petrified ears are an uncommon finding which is nothing but hardening of articular cartilage as a result of calcification. Various endocrinopathies such as Addison’s disease, diabetes, hypothyroidism may cause this calcification of ears (12). Usually, the presence of ear calcification is a useful sign as it may precede the development of endocrinopathies by many years. Further, mechanical trauma and frost bite are other two major causes.

The second most common incident STC is triticeous cartilage calcification. These are tiny oval shaped cartilage located at the lateral border of thyrohyoid membrane just below hyoid bone in C3-C4 region. Though triticeous cartilage doesn’t pose a significant and specific function, calcification of it is a usual thing and said to occur in same pattern as thyroid cartilage (13). In our study, significant bilateral calcification was noted with a female predilection. Calcification usually begins in the second decade of life and terminated at the elderly age. In our study, most of the calcifications were partially calcified irrespective of the age group. This denoted triticeous cartilage calcification is independent of aging, in accordance with findings of Hatley *et al*. (14). Though triticeous cartilage calcification is of less clinical significance being asymptomatic and the cartilage having no known functions, it should be investigated for the presence of dysphagia, odynophagia (15) and any endocrinopathies.

Another noteworthy STC sialolith, they are calcific deposits in the gland formed around a central nidus. In this study about 90% of sialolith were encountered in submandibular gland. This finding is in accordance with findings of Lustman *et al*. (16). Radiographically, they had a homogenous calcification with a laminated appearance. Though sialolith are of less clinical significance when they are of small size, they have potential to be the basis of various salivary gland pathologies such as sialadenitis which may result in total excision of the gland. Further about 20% of submandibular gland stones and 40% of parotid gland stones are unseen on routine plain films (17). Thus, an additional sialogram should be performed followingly to rule out presence of additional sialolith.

Tonsilolith showed significant completely calcified structures with male predilection and unilateral involvement. Most of the calcified structures were spherical (68%) in shape followed by oval and a significant multiple calcifications (*p*=.009) were found to the highest of 8 calcifications per side. A mean size of 1*1 cm was observed. Tonsilolith was observed in a broad age range while adult and middle-aged persons were most commonly affected. Presence of tonsil stones may indicate the presence of long standing or chronic tonsilitis which warrants definitive treatment.

Most prevalent STC is the stylohyoid ligament calcification. Bilateral stylohyoid ligament calcifications were common with a mean size of 2.5 ± 0.5 cm, with comparatively more partial calcifications and female predilection. When looking into region, occurrence in L, G region was most commonly noted followed by A, G regions in bilateral cases. Region L was most commonly involved in unilateral cases. In most of the instances this condition is asymptomatic, only 28% of cases show symptoms (18). The symptoms vary from a simple foreign body sensation in throat to Eagle’s syndrome. Severe calcification of the ligament can be an indicator for underlying disturbance in calcium homeostasis resulting from endocrinopathies. The longest of the observed stylohyoid ligament calcification was 8 cm long with a width of 1.5 cm extending all the way from the styloid process to hyoid bone bilaterally (48 years, male). Likewise, calcification of the stylomandibular ligament was also noted which mostly occurred unilaterally with female predilection.

An interesting case of cervical lymph node calcification of size 2.5*3 cm was observed in our study in a patient of age 17 years. When checking the medical background of the patient, previous tuberculous infection was identified. Tuberculous lymphadenitis- scrofula is one of the primary causes for dystrophic lymph node calcification (19). Other possible causes include cancer metastasis, chronic infection. In our analysis, most of the lymph node calcifications in oral and maxillofacial region were submandibular of size 1.5*1 cm, having irregular completely calcified features. The mean age of incidence was 51 years with a female predilection. Usually, they are asymptomatic and no treatment is needed. Whereas in patients with cancer, resection of the lymph nodes is necessary to prevent further spread and recurrence (20).

Other STC identified in this study include antrolith, rhinolith, calcinosis cutis, TMJ calcifications and myositis ossificans. These calcifications had a strong unilateral involvement with a female predilection. They were mostly partially calcified structures of size 1*1 cm. Antrolith and Rhinolith are calcified masses present in the maxillary sinus and nasal cavity respectively. Radiographically they appeared round to ovoid shape and antrolith was accompanied with the features of maxillary sinusitis, which includes mucosal thickening. Patients with antrolith and rhinolith may be asymptomatic until incidental radiographic identification. Some of the symptoms associated include difficulty breathing, purulent discharge, anosmia and oroantral fistula with palatal or nasal septal perforation (21). TMJ calcifications are comparatively rare and when identified proper care should be taken as they have high chances to progress into severe joint deformities. Two possible causes for TMJ calcifications include synovial chondromatosis and calcium pyrophosphate dihydrate deposition (22). In our examination, they appeared as small multiple partially calcified structures in the region between mandibular coronoid process and articular eminence. Mean size was 0.5*0.5 cm.

## Conclusions

PR is an essential diagnostic tool used in routine dental procedures which also has an added advantage of early diagnosis of diseases with the help of STC present. Soft tissue calcifications in the oral and maxillofacial regions are relatively common and are also of great importance. Majority of the calcifications reported in this study were stylohyoid ligament calcification followed by calcified atherosclerotic plaque, sialolith, triticeous cartilage calcification. As dentists we play an important role in early identification of undiagnosed systemic disease with incidental detection of STC present in routine PR. The early detection is also important to prevent further progression of disease thus reducing mortality and morbidity of patients. In order to achieve this a thorough interpretation of all routine radiographs extending beyond the area of interest is decisive. Few crucial STC reported in this study include calcified atherosclerotic plaque of carotid and superficial temporal artery, petrified ear, oro-pharyngeal calcification, monckeberg atherosclerosis.
